# Geographic disparities of dietary inflammatory index and its association with hypertension in middle-aged and elders in China: results from a nationwide cross-sectional study

**DOI:** 10.3389/fnut.2024.1355091

**Published:** 2024-03-07

**Authors:** Weihua Dong, Qingqing Man, Jian Zhang, Zhen Liu, Weiyi Gong, Liyun Zhao, Pengkun Song, Gangqiang Ding

**Affiliations:** ^1^Department of Geriatric and Clinical Nutrition, Chinese Center for Diseases Control and Prevention, National Institute for Nutrition and Health, Beijing, China; ^2^Key Laboratory of Trace Elements and Nutrition of National Health Commission, Beijing, China; ^3^Department of Nutrition Surveillance, Chinese Center for Diseases Control and Prevention, National Institute for Nutrition and Health, Beijing, China

**Keywords:** dietary inflammatory index, hypertension, cross-sectional study, spatial statistical analysis, restricted cubic spline, LASSO regression

## Abstract

**Background:**

Geographic distribution of dietary inflammatory index (DII) in China has not been thoroughly evaluated and evidence on the association between DII and hypertension among Chinese middle-aged and older population was inadequate.

**Objective:**

To investigate the geographic disparities of DII and its association with hypertension among Chinese middle-aged and elders.

**Methods:**

Data was from the China Adults Chronic Diseases and Nutrition Surveillance (CACDNS 2015) for middle-aged and older participants. The DII for each participant was determined through a combination of 3 days 24 h dietary recall interviews and a food frequency questionnaire. Spatial analysis was employed to investigate the geographic distribution of DII in China. Restricted cubic spline models and binary logistic regression analysis were used to assess the relationship between DII and hypertension. The least absolute shrinkage and selection operator (LASSO) regression was applied for identifying key hypertension-related factors, which was then included in the establishment of a risk prediction nomogram model, with the receiver operating characteristic (ROC) curve and decision curve analysis (DCA) being built to evaluate its discriminatory power for hypertension.

**Results:**

A total of 52,087 middle-aged and older participants were included in the study, among whom 36.6% had hypertension. it revealed that a clear spatial correlation in the national distribution of DII scores (Moran I: 0.252, *p* = 0.001), with higher DII scores concentrated in the northwest region and lower DII scores concentrated in the southeast region. Hypertensive participants had higher DII scores compared to those without hypertension (OR: 1.507 vs. 1.447, *p* = 0.003). Restricted cubic spline models and binary logistic regression analysis demonstrated a positive association between DII and hypertension after adjusting for potential confounding factors. There was a significant increasing trend in the proportion of hypertensive individuals as DII scores increase (*p* for trend = 0.004). The nomogram model, constructed using key factors identified through LASSO regression, demonstrated a robust discriminative capacity, with an area under the curve (AUC) of 73.2% (95% CI, 72.4–74.0%). Decision curve analysis confirmed the reliability and effectiveness of the nomogram model. Sensitivity analysis conducted within the subpopulation aged under 45 years yielded results consistent with the primary analysis.

**Conclusion:**

In Chinese adults middle-aged and older, geographic disparities in dietary inflammatory potential are notable, with lower levels observed in the southeastern coastal regions of China and higher levels in the northwestern regions. Meanwhile, there is a positive association between the inflammatory potential of the diet and hypertension. Additional research is needed to investigate regional disparities in dietary inflammatory potential and pinpoint specific dietary patterns associated with lower inflammation.

## 1 Introduction

Hypertension poses a significant global threat to public health (1, 2), resulting in a considerable number of non-communicable deaths, accounting for almost 20% of all deaths in 2019 (3). The World Health Organization estimates that more than 1.3 billion adults worldwide are affected by hypertension (4), which places a substantial burden on both individual health and socio-economic development. Report from six national surveys of China revealed that there were approximately 274 million hypertensive patients aged 18–69 years in 2018, with a standardized prevalence rate of 24.7% (5).

The rapid social and economic development in China has brought about lifestyle changes that can negatively impact residents’ health. Un-healthy lifestyles such as inadequate physical activity, insufficient sleep, and irregular eating habits will increase the risk of hypertension (6, 7). Particularly, poor diet, including heavy alcohol consumption, excessive salt intake, and a potassium-deficient diet, is closely associated with hypertension (8). Dietary patterns linked to CRP have been identified as crucial risk factors for hypertension and metabolic syndrome (9). Elevated levels of DII and CRP are associated with an increased risk of developing hypertension and metabolic syndrome (10). Conversely, certain healthy dietary patterns, such as the DASH diet and plant-based diets, have been found to reduce blood pressure and lower inflammatory marker levels (11, 12). Inflammation plays a crucial role in hypertension, as evidenced by numerous studies demonstrating elevated systemic inflammation levels in hypertensive patients, which are closely linked to arterial stiffness (13, 14).

In recent studies, the focus has been on the changes in the level of inflammation caused by diet and its ultimate impact on hypertension. Therefore, better understanding the relationship between hypertension risk and dietary habits is essential for preventing its occurrence and progression. The Dietary Inflammatory Index (DII) has been developed to reflect the inflammatory potential of the diet and score an individual’s diet on a continuous spectrum from anti-inflammatory to pro-inflammatory (15). A higher DII score indicates a pro-inflammatory diet, while a lower score reflects an anti-inflammatory diet. The DII scores are standardized based on global dietary intake, allowing them to be applied across various cultures and dietary patterns. However, limited studies had been conducted on the relationship between DII and hypertension specifically in the Chinese population, particularly among the middle-aged and older population. Therefore, it remains uncertain whether there is a correlation between DII and the prevalence of hypertension among the Chinese adults 45 years and above. Particularly, as an essential dietary factor affecting hypertension, the study of the relationship between DII and hypertension risk, as well as evaluating the potential of DII in preventing and managing hypertension, can significantly advance primary prevention efforts in hypertension control. Additionally, it can offer valuable insights for health management strategies in the middle-aged population. In light of these objectives, this study conducted a cross-sectional study based on the China Adults Chronic Diseases and Nutrition Surveillance (2015) to further investigate the association between hypertension and DII.

## 2 Methods

### 2.1 Data source and survey population

The data used in this study was from the China Adults Chronic Diseases and Nutrition Surveillance (2015) conducted throughout mainland China’s 31 provinces, autonomous regions, and municipalities (excluding Taiwan, Hong Kong, and Macao). Multi-stage stratified cluster random sampling method was applied to collect participants aged 18 years and above. The detailed description of the sampling procedure was described in reference (16). This study was approved by the Ethics Committee of the Chinese Center for Disease Control and Prevention (Approval No. 201519-B), and informed consent was obtained from all participants prior to their enrollment. Comprehensive information on participants’ basic demographics, lifestyles, diets, and health statuses was collected through household and personal questionnaires, body measurements, dietary surveys, and laboratory tests. This study specifically focused participants middle-aged and older (age ≥45 years).

**Figure 1:**
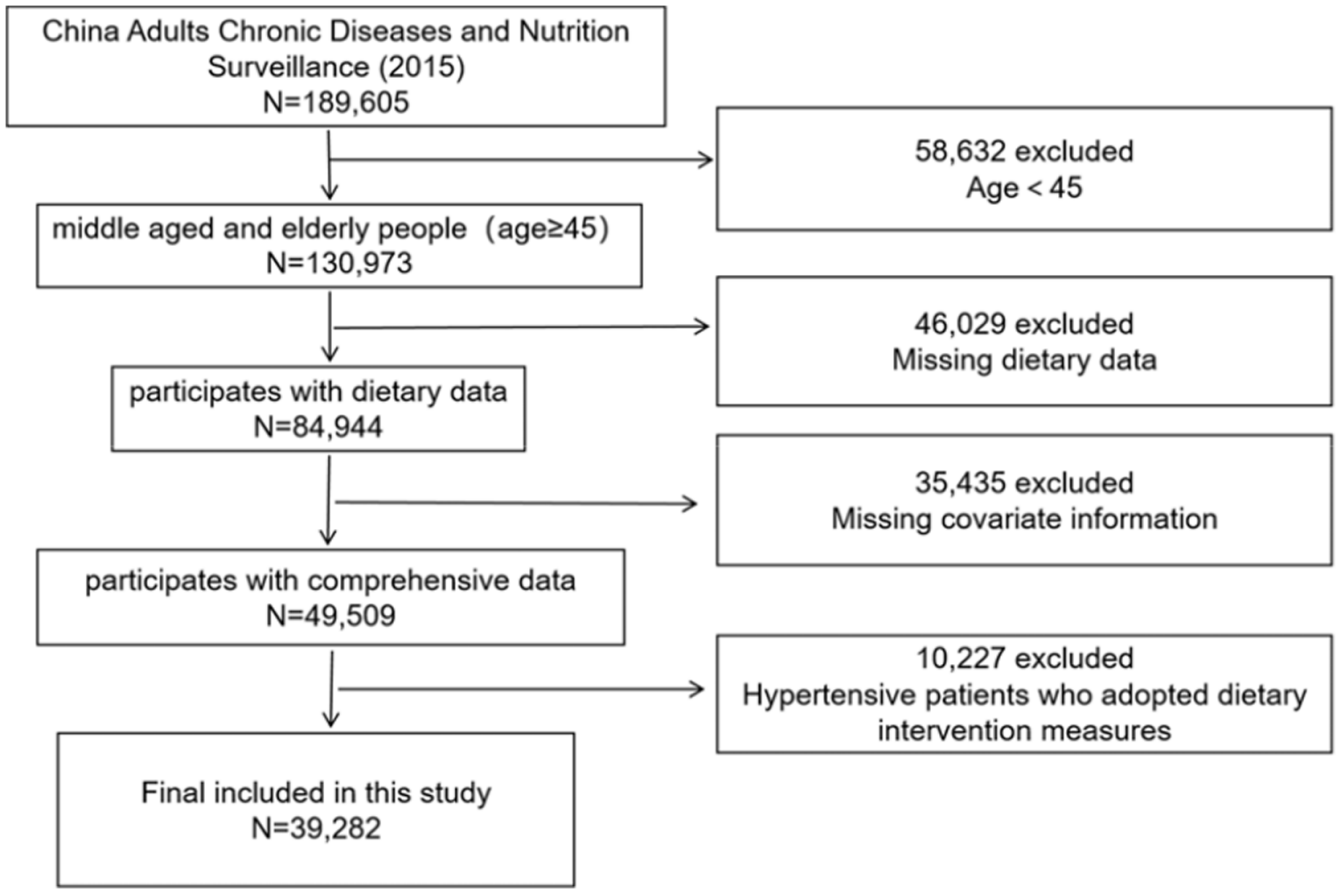
Flow diagram of participants in the study.

The study initially excluded research subjects without dietary data and enrolled all participants aged 45 and above with dietary information (*n* = 52,087). In the subsequent analysis of the association between DII and hypertension, individuals who adopt interventions such as diet and exercise will be removed to mitigate the influence of subjective selection on the study’s integrity. Furthermore, participants with missing crucial demographic data will be excluded. Ultimately, 39,282 individual were involved in this study (Figure 1).

### 2.2 General information collection and dietary nutrients assessment

A standardized questionnaire was used to collect general demographic information, lifestyle, health status, and physical activity data through face-to-face interviews conducted by trained investigators at participants’ residencies. The investigators provided assistance to the participants to complete the survey questionnaire. The dietary survey comprised 3 days 24 h dietary recalls and a food frequency questionnaire (FFQ) administered via personal interviews. Anthropometric measurements, including height, weight, waist circumference, and blood pressure were collected using standard methods (17) and certified measuring instruments (TZG height altimeter and Tanita HD-390 electronic weight scale) approved and designated by the National Project Team, with readings that were accurate to 0.1 cm and 0.1 kg, respectively. When height and weight measurements were obtained, participants were asked to remove their shoes and hats and don lightweight clothing. Serum fasting glucose were measured enzymically using an automatic biochemical analyzer (Hitachi 7600, Tokyo, Japan). Glycated hemoglobin (HbA1c) was determined by high-performance liquid chromatography (HPLC) using Trinity Biotech, Premier Hb9210 (Dublin, Ireland).

### 2.3 Definition of hypertension

Hypertension was defined as follows: (1) average systolic blood pressure (SBP) ≥140 mmHg and average diastolic blood pressure (DBP) ≥90 mmHg, (2) self-reported hypertension, (3) individuals with prescribed anti-hypertensive medications, (4) individuals who adopt interventions such as diet and exercise. The criteria of 140/90 mmHg refers to the guideline of the 2018 Chinese guidelines for the management of hypertension (18).

### 2.4 Dietary inflammation index calculation and their geographic variations

The 3 days 24 h dietary recalls were used to collect detailed information regarding all food consumed by participants in their homes and outside over three consecutive days (two weekdays and one weekend day), including staple foods, non-staple foods, snacks, fruits, and beverages. Participants were also asked to provide information on food frequency and consumption amounts for given foods in the past 12 months or 1 month using the FFQ. All food intake, including breakfast, lunch, dinner, and snack times, was documented during the interview period.

The DII was calculated based on dietary data obtained from a 3 days 24 h meal review survey and FFQ, using the Chinese Food Composition Table, which provides nutrition data for over 2,000 foods and ingredients. By integrating with the Chinese Food Composition Database, the 3 days 24 h dietary recall can provide the daily intake of various nutrients for each participant. Additionally, through the conversion of intake frequency and amount, the FFQ can determine the daily alcohol intake of each participant.

The DII assesses the inflammation effects of dietary consumption from 45 nutrients, and calculates an individual’s overall inflammatory potential score based on these nutrients. Each nutrient’s *z*-score was first obtained as (daily mean intake reported − global daily mean intake)/standard deviation, and then transformed into a percentile score to minimize “right skewing.” The percentile value for each nutrient was multiplied by its respective “overall inflammatory effect score” to obtain a “nutrient-specific DII score,” which in turn was added up to achieve an individual’s “overall DII score.”

Previous studies had shown that the DII remain valid even if fewer than 30 nutrients were used for calculation (19, 20). In this study, a total of 23 nutrients were considered in the calculation of the DII, including total energy, protein, fat, carbohydrate, fiber, cholesterol, vitamin A, carotene, thiamin, riboflavin, vitamin B_6_, vitamin B_12_, folic acid, niacin, vitamin C, vitamin E, Mg, Fe, Zn, Se, monounsaturated fatty acids (MUFA), polyunsaturated fatty acids (PUFA), and alcohol. The participants were divided into four groups according to the quartiles of DII scores (Q1: −4.379 to 0.307, lowest group; Q2: 0.308 to 1.456; Q3: 1.457 to 2.176; Q4: 2.177 to 4.285, highest group). In addition, to describe the regional differences in DII, this study also calculated the DII for research populations in 31 provinces, which was used for spatial analysis and displayed by choropleth maps. The spatial dependencies of DII were investigated through an exploratory analysis that employed global and local spatial autocorrelation techniques to assess the DII distribution. The principal measure of spatial correlation, the global Moran’s I (GMI) statistic, was employed in this study. GMI values, which range from −1 to 1, serve as indicators: a GMI near −1 indicates dispersed adherence, while a GMI close to 1 signifies clustered adherence. A GMI of 0 denotes randomly distributed adherence. Significantly *p*-values (*p* < 0.05) linked to GMI values can invalidate the null hypothesis, suggesting spatial autocorrelation. Further analysis through local spatial autocorrelation techniques highlighted significant spatial clusters. These include high-high and low-low clusters, which represent regions of similar DII scores being geographically proximate. Conversely, low-high and high-low outliers were identified, indicating regions where dissimilar DII scores were found in close proximity. Additionally, hotspot analysis, incorporating spatial weighting, normalization of values, and *z*-score calculations, was utilized to discern areas of spatial concentration. This analysis revealed hotspots (areas with positive *z*-scores) and coldspots (areas with negative *z*-scores), providing deeper insights into regions with, respectively, higher or lower clustered DII scores.

### 2.5 Covariant index

In this study, the covariates included age categorized into three subgroups (45–59 years, 60–74 years, and ≥ 75 years), gender (male or female), educational level (below junior high school, junior high school, and high school or above), region (urban or rural), adequate physical activity (yes or no), marital status (married or other), smoking (yes or no), alcohol consumption (yes or no), BMI (underweight BMI <18.5 kg/m^2^, normal weight BMI 18.5–23.9 kg/m^2^, overweight BMI 24.0–27.9 kg/m^2^, and obese BMI ≥28.0 kg/m^2^) (21), family history of chronic diseases (yes or no), central obesity (yes or no), diabetes mellitus (yes or no) and daily sleep duration (<6 h/day, 6–8 h/day, or ≥8 h/day). “Other” marital status includes unmarried, divorced, and widowed individuals. Smoking is defined as having smoked at least once in the past 30 days, while drinking is defined as having consumed alcohol at least once in the past year. Adequate physical activity is defined as engaging in moderate or above physical activity more than 150 min per typical week. BMI was calculated by dividing weight in kilograms by height in meters squared (kg/m^2^). Central obesity was defined as a waist circumference of ≥90 cm for men and ≥85 cm for women. Diabetes mellitus was diagnosed according to the ADA 2010 criteria (FPG level ≥7.0 mmol/L and/or HbA1c concentration ≥6.5%) (22).

### 2.6 Statistical analysis

Statistical analyses were conducted using R 4.2.2 and SAS 9.4 (SAS Institute, Cary, NC, United States). Spatial analysis was performed using ArcGIS Desktop version 10.8 (ESRI, Inc., Redlands, CA, United States). Categorical variables were expressed as counts (percentages) and were compared using the chi-squared test. Continuous variables were expressed as mean (standard error, SE) if data presented a normal distribution and were compared using analysis of variance (ANOVA), or expressed as medians (25th, 75th), if data presented a skewed distribution and were compared by the Wilcoxon rank sum test. After categorizing the DII scores into quartiles, a binary logistic regression model was used to examine the relationship between DII and hypertension, while adjusting for potential confounding covariates (age, gender, educational level, physical activity, smoking, drinking, BMI, central obesity, diabetes, and family history of chronic diseases). In addition, a restricted cubic spline model was applied to evaluate the dose–response relationship between DII and hypertension, adjusting for the above-mentioned covariates. Global and local Moran’s I were used to detect spatial autocorrelation and local heterogeneity of dietary patterns. DII by province were calculated and shown with choropleth maps. Statistical significance was defined as two-sided *p*-values less than 0.05.

## 3 Results

### 3.1 Baseline characteristics of the study participants

In this study, a total of 52,087 individuals aged 45 and above with available dietary data were included, consisting of 25,269 males (48.51%) and 26,818 females (51.49%). Among them, 19,048 individuals (36.6%) had hypertension (Table 1). After excluding participants who adopt interventions such as diet and exercise, as well as those with missing key covariates, a total of 39,282 individuals were finally included in this study, with 6,133 individuals having hypertension (15.6%). Among them, 19,135 (49.2%) were male, 15,132 (38.5%) were from urban areas, and 33,709 (94.2%) were married. The median age of all participants was 58.1 years (Table 2).

**Table 1:** Baseline characteristics of the population middle-aged and older with dietary data.

	ALL	Male	Female	*p-overall*
	*N* = 52,087	*N* = 25,269	*N* = 26,818	
Age (median, Q1, Q3, year)	59.3 [51.8; 66.2]	60.0 [52.1; 66.8]	58.8 [51.6; 65.6]	<0.001
Age group (*n*, %)				<0.001
45–59 years	27,270 (52.35)	12,692 (50.23)	14,578 (54.36)	
60–74 years	21,053 (40.42)	10,558 (41.78)	10,495 (39.13)	
≥75 years	3,764 (7.23)	2019 (7.99)	1745 (6.51)	
Region (*n*, %)				<0.001
Urban	21,097 (40.50)	9,983 (39.51)	11,114 (41.44)	
Rural	30,990 (59.50)	15,286 (60.49)	15,704 (58.56)	
Han ethnicity (*n*, %)	46,788 (89.83)	22,709 (89.87)	24,079 (89.79)	0.767
Adequate physical activity (*n*, %)	36,193 (69.49)	16,514 (65.35)	19,679 (73.38)	<0.001
Educational level (*n*, %)				<0.001
Below junior high school	29,900 (57.40)	12,029 (47.60)	17,871 (66.64)	
Junior high school	20,252 (38.88)	12,036 (47.63)	8,216 (30.64)	
Senior high school or above	1935 (3.71)	1,204 (4.76)	731 (2.73)	
Marital status (*n*, %)				<0.001
Other status	3,361 (6.45)	1,096 (4.34)	2,265 (8.45)	
Having a partner	48,726 (93.55)	24,173 (95.66)	24,553 (91.55)	
Smoking (*n*, %)	14,134 (27.14)	13,205 (52.26)	929 (3.46)	<0.001
Drinking (*n*, %)	18,071 (34.69)	14,055 (55.62)	4,016 (14.98)	<0.001
Sleep time (median, Q1, Q3, h/day)	8.00 [7.00; 8.00]	8.00 [7.00; 8.00]	8.00 [6.50; 8.17]	<0.001
Sleep time group (*n*, %)				<0.001
<6 h/day	5,142 (9.87)	2,116 (8.37)	3,026 (11.28)	
6–8 h/day	34,019 (65.31)	16,953 (67.09)	17,066 (63.64)	
>8 h/day	12,926 (24.82)	6,200 (24.54)	6,726 (25.08)	
BMI (median, Q1, Q3, kg/m^2^)	24.0 [21.7; 26.5]	23.8 [21.5; 26.2]	24.2 [21.9; 26.8]	<0.001
BMI group (*n*, %)				<0.001
Under weight	2,863 (5.50)	1,449 (5.73)	1,414 (5.27)	
Normal weight	22,946 (44.05)	11,702 (46.31)	11,244 (41.93)	
Overweight	18,863 (36.21)	9,034 (35.75)	9,829 (36.65)	
Obesity	7,415 (14.24)	3,084 (12.20)	4,331 (16.15)	
Family history of chronic diseases (*n*, %)	22,295 (42.80)	10,492 (41.52)	11,803 (44.01)	<0.001
Fasting blood-glucose (median, Q1, Q3, mmol/L)	5.26 [4.83; 5.78]	5.27 [4.82; 5.80]	5.26 [4.85; 5.76]	0.684
Diabetes mellitus (*n*, %)	6,125 (11.76)	2,893 (11.45)	3,232 (12.05)	0.034
Systolic blood pressure (median, Q1, Q3, mmHg)	136.67 [124.00; 152.33]	136.33 [124.33; 151.00]	137.00 [124.00; 153.33]	<0.001
Diastolic blood pressure (median, Q1, Q3, mmHg)	79.67 [72.33; 87.33]	81.00 [74.00; 88.67]	78.33 [71.00; 85.67]	<0.001
Hypertension (*n*, %)	19,048 (36.57)	9,541 (37.76)	9,507 (35.45)	<0.001
Central obesity (*n*, %)	17,997 (34.55)	7,589 (30.03)	10,408 (38.81)	<0.001

**Table 2:** Characteristics of subjects according to hypertension status.^a^

	ALL	Non-hypertension	Hypertension	*p-overall*
	*N* = 39,282	*N* = 33,149	*N* = 6,133	
Male (*n*, %)	19,315 (49.2)	15,762 (47.5)	3,553 (57.9)	<0.001
Age (Median, Q1, Q3, year)	58.1 [51.0; 64.9]	58.0 [50.9; 64.9]	58.5 [51.8; 65.2]	<0.001
Age group (*n*, %)				0.012
45–59 years	22,408 (57.0)	19,014 (57.4)	3,394 (55.3)	
60–74 years	14,556 (37.1)	12,185 (36.8)	2,371 (38.7)	
≥75 years	2,318 (5.90)	1950 (5.88)	368 (6.00)	
Urban (*n*, %)	15,132 (38.5)	12,991 (39.2)	2,141 (34.9)	<0.001
Han ethnicity	35,128 (89.4)	29,730 (89.7)	5,398 (88.0)	
Adequate physical activity (*n*, %)	27,994 (71.3)	23,825 (71.9)	4,169 (68.0)	<0.001
Educational level (*n*, %)				<0.001
Below junior high school	22,542 (57.4)	19,045 (57.5)	3,497 (57.0)	
Junior high school	15,354 (39.1)	12,876 (38.8)	2,478 (40.4)	
Senior high school or above	1,386 (3.53)	1,228 (3.70)	158 (2.58)	
Marital status (*n*, %)				0.002
Other status	2,273 (5.79)	1865 (5.63)	408 (6.65)	
Having a partner	37,009 (94.2)	31,284 (94.4)	5,725 (93.3)	
Smoking (*n*, %)	11,184 (28.5)	9,260 (27.9)	1924 (31.4)	<0.001
Drinking (*n*, %)	14,173 (36.1)	11,558 (34.9)	2,615 (42.6)	<0.001
Sleep time (median, Q1, Q3, h/day)	8.00 [7.00; 8.00]	8.00 [7.00; 8.00]	8.00 [7.00; 8.00]	0.234
Sleep time group (*n*, %)				0.543
<6 h/day	3,709 (9.44)	3,133 (9.45)	576 (9.39)	
6–8 h/day	26,017 (66.2)	21,986 (66.3)	4,031 (65.7)	
>8 h/day	9,556 (24.3)	8,030 (24.2)	1,526 (24.9)	
BMI (Median, Q1, Q3, kg/m^2^)	23.8 [21.6; 26.1]	23.6 [21.4; 25.8]	24.9 [22.6; 27.3]	<0.001
BMI group (*n*, %)				<0.001
Underweight	1,526 (3.88)	1,394 (4.21)	132 (2.15)	
Normal weight	19,216 (48.9)	16,942 (51.1)	2,274 (37.1)	
Overweight	13,843 (35.2)	11,333 (34.2)	2,510 (40.9)	
Obesity	4,697 (12.0)	3,480 (10.5)	1,217 (19.8)	
Family history of chronic diseases (*n*, %)	15,099 (38.4)	12,647 (38.2)	2,452 (40.0)	0.007
Central obesity (*n*, %)	12,163 (31.0)	9,563 (28.8)	2,600 (42.4)	<0.001
Fasting blood-glucose (median, Q1, Q3, mmol/L)	5.24 [4.83; 5.71]	5.22 [4.82; 5.68]	5.35 [4.91; 5.87]	<0.001
Diabetes mellitus (*n*, %)	3,551 (9.04)	2,847 (8.59)	704 (11.5)	<0.001

a Participants who adopt interventions such as diet and exercise, as well as those with missing key covariates were excluded.

Table 3 showed the DII scores and its nutrients source by hypertensive status. Negative scores indicated anti-inflammatory diets, while positive scores represented pro-inflammatory diets. The overall median of DII score was 1.457, 1.507 and 1.447 in those with and without hypertension, respectively. The DII score of hypertensive participants was statistically significantly higher compared to non-hypertensive participants (*p* = 0.003). The differences in DII scores between the two groups primarily manifested in several dietary components and nutrients, including protein, dietary fiber, cholesterol, vitamin A, carotene, thiamin, riboflavin, folate, niacin, vitamin C, magnesium, zinc, selenium, alcohol, and MUFA.

**Table 3:** Comparison of each component of DII scores between individuals with and without hypertension.

	ALL	Non-hypertension	Hypertension	*p-overall*
Total score	1.457 [0.308; 2.177]	1.447 [0.289; 2.174]	1.507 [0.406; 2.190]	0.003
Energy (kcal)	0.01 [−0.15; 0.17]	0.01 [−0.15; 0.17]	0.01 [−0.15; 0.17]	0.767
Protein (g)	−0.016 [−0.021; 0.009]	−0.016 [−0.021; 0.009]	−0.017 [−0.021; 0.009]	0.001
Fat (g)	0.077 [−0.199; 0.290]	0.079 [−0.198; 0.290]	0.070 [−0.205; 0.289]	0.12
Carbohydrate (g)	−0.001 [−0.090; 0.095]	−0.001 [−0.090; 0.095]	−0.002 [−0.090; 0.095]	0.987
Fiber (g)	0.608 [0.345; 0.652]	0.607 [0.339; 0.652]	0.613 [0.376; 0.653]	0.002
Cholesterol (mg)	−0.110 [−0.110; −0.032]	−0.110 [−0.110; −0.028]	−0.110 [−0.110; −0.047]	<0.001
Vitamin A (RE)	0.314 [0.185; 0.355]	0.312 [0.181; 0.354]	0.323 [0.206; 0.359]	<0.001
β-carotene (μg)	0.485 [0.185; 0.548]	0.482 [0.176; 0.547]	0.504 [0.237; 0.551]	<0.001
Thiamin (mg)	0.078 [0.053; 0.088]	0.078 [0.053; 0.088]	0.079 [0.054; 0.089]	0.022
Riboflavin (mg)	0.053 [0.040; 0.058]	0.052 [0.040; 0.058]	0.053 [0.042; 0.059]	<0.001
Vitamin B_6_ (mg)	0.348 [0.347; 0.348]	0.348 [0.347; 0.348]	0.348 [0.347; 0.348]	0.031
Vitamin B_12_ (μg)	−0.100 [−0.100; −0.100]	−0.100 [−0.100; −0.100]	−0.100 [−0.100; −0.100]	0.256
Folate (μg)	0.143 [−0.012; 0.182]	0.142 [−0.016; 0.182]	0.150 [0.013; 0.183]	<0.001
Niacin (mg)	0.16 [0.08; 0.20]	0.16 [0.08; 0.20]	0.17 [0.09; 0.20]	<0.001
Vitamin C (mg)	0.243 [−0.095; 0.372]	0.238 [−0.104; 0.371]	0.266 [−0.050; 0.379]	<0.001
Vitamin E (mg)	−0.419 [−0.419; −0.419]	−0.419 [−0.419; −0.419]	−0.419 [−0.419; −0.419]	0.156
Mg (mg)	0.071 [−0.195; 0.248]	0.069 [−0.198; 0.247]	0.078 [−0.178; 0.252]	0.018
Fe (mg)	0.031 [0.016; 0.032]	0.031 [0.016; 0.032]	0.031 [0.015; 0.032]	0.006
Zn (mg)	−0.062 [−0.297; 0.205]	−0.065 [−0.298; 0.203]	−0.047 [−0.294; 0.213]	0.002
Se (μg)	0.133 [0.030; 0.169]	0.132 [0.030; 0.168]	0.136 [0.033; 0.169]	0.03
Alcohol (g)	0.278 [−0.018; 0.278]	0.278 [0.199; 0.278]	0.278 [−0.278; 0.278]	<0.001
MUFA (g)	−0.009 [−0.009; −0.009]	−0.009 [−0.009; −0.009]	−0.009 [−0.009; −0.009]	<0.001
PUFA (g)	−0.337 [−0.337; −0.337]	−0.337 [−0.337; −0.337]	−0.337 [−0.337; −0.337]	0.7

Table 4 showed the distribution of participant characteristics according to the quartiles of DII, which range from −4.379 to 4.285. As DII score quartiles increased, there was a significant increasing trend observed in the prevalence of hypertension among the participants (*p* = 0.004). Moreover, there was a significant increasing trend in the proportion of females and in the levels of systolic and diastolic blood pressure, whereas the proportion of males and age demonstrated a decreasing trend (*p* < 0.01).

**Table 4:** Characteristics of subjects according to DII quartile distribution.

	Q1 (−4.379 to 0.307)	Q2 (0.308–1.456)	Q3 (1.457–2.176)	Q4 (2.177–4.285)	*p-overall*	*p-trend*
*N*	9,821	9,820	9,821	9,820		
Male (*n*, %)	4,947 (50.4)	5,072 (51.6)	4,996 (50.9)	4,300 (43.8)	<0.001	<0.001
Age (median, Q1, Q3, year)	59.1 [51.7; 65.0]	58.4 [51.4; 65.0]	57.5 [50.9; 64.6]	57.2 [50.3; 65.1]	<0.001	<0.001
Age group (*n*, %)					<0.001	<0.001
45–59 years	5,250 (53.5)	5,472 (55.7)	5,836 (59.4)	5,850 (59.6)		
60–74 years	4,079 (41.5)	3,799 (38.7)	3,408 (34.7)	3,270 (33.3)		
≥75 years	492 (5.01)	549 (5.59)	577 (5.88)	700 (7.13)		
Urban (*n*, %)	4,675 (47.6)	3,972 (40.4)	3,453 (35.2)	3,032 (30.9)	<0.001	<0.001
Nation (*n*, %)					<0.001	<0.001
Han ethnicity	9,164 (93.3)	8,949 (91.1)	8,745 (89.0)	8,270 (84.2)		
Other ethnicities	657 (6.69)	871 (8.87)	1,076 (11.0)	1,550 (15.8)		
Adequate physical activity (*n*, %)	7,112 (72.4)	7,105 (72.4)	6,983 (71.1)	6,794 (69.2)	<0.001	<0.001
Educational level (*n*, %)					<0.001	<0.001
Below junior high school	4,847 (49.4)	5,531 (56.3)	5,745 (58.5)	6,419 (65.4)		
Junior high school	4,343 (44.2)	3,961 (40.3)	3,827 (39.0)	3,223 (32.8)		
Senior high school or above	631 (6.43)	328 (3.34)	249 (2.54)	178 (1.81)		
Marital status (*n*, %)					<0.001	<0.001
Other status	504 (5.13)	522 (5.32)	559 (5.69)	688 (7.01)		
Having a partner	9,317 (94.9)	9,298 (94.7)	9,262 (94.3)	9,132 (93.0)		
Smoking (*n*, %)	2,788 (28.4)	2,988 (30.4)	2,974 (30.3)	2,434 (24.8)	<0.001	<0.001
Drinking (*n*, %)	4,397 (44.8)	3,934 (40.1)	3,716 (37.8)	2,126 (21.6)	<0.001	<0.001
Sleep time (median, Q1, Q3, h/day)	8.00 [7.00; 8.00]	8.00 [7.00; 8.00]	8.00 [7.00; 8.50]	8.00 [7.00; 8.50]	<0.001	<0.001
Sleep time group (*n*, %)					<0.001	0.017
<6 h/day	846 (8.61)	953 (9.70)	946 (9.63)	964 (9.82)		
6–8 h/day	6,757 (68.8)	6,502 (66.2)	6,395 (65.1)	6,363 (64.8)		
>8 h/day	2,218 (22.6)	2,365 (24.1)	2,480 (25.3)	2,493 (25.4)		
BMI (median, Q1, Q3, kg/m^2^)	23.9 [21.8; 26.1]	23.7 [21.6; 26.1]	23.7 [21.5; 26.1]	23.7 [21.4; 26.1]	0.005	0.001
BMI group (*n*, %)					0.002	0.078
Underweight	338 (3.44)	358 (3.65)	403 (4.10)	427 (4.35)		
Normal weight	4,751 (48.4)	4,831 (49.2)	4,802 (48.9)	4,832 (49.2)		
Overweight	3,595 (36.6)	3,483 (35.5)	3,404 (34.7)	3,361 (34.2)		
Obesity	1,137 (11.6)	1,148 (11.7)	1,212 (12.3)	1,200 (12.2)		
Family history of chronic diseases (*n*, %)	4,420 (45.0)	3,943 (40.2)	3,601 (36.7)	3,135 (31.9)	<0.001	<0.001
Central obesity (*n*, %)	3,051 (31.1)	3,046 (31.0)	3,040 (31.0)	3,026 (30.8)	0.983	0.695
Fasting blood-glucose (median, Q1, Q3, mmol/L)	5.26 [4.87; 5.73]	5.24 [4.84; 5.72]	5.23 [4.83; 5.70]	5.22 [4.80; 5.69]	<0.001	<0.001
Diabetes mellitus (*n*, %)	932 (9.49)	907 (9.24)	850 (8.65)	862 (8.78)	0.141	0.036
Sbp (median, Q1, Q3, mmHg)	133 [122; 146]	134 [123; 147]	135 [123; 148]	134 [123; 149]	<0.001	<0.001
Dbp (median, Q1, Q3, mmHg)	78.6 (12.1)	79.0 (12.0)	79.2 (12.2)	79.1 (12.1)	0.002	0.003
Hypertension (*n*, %)	1,426 (14.5)	1,547 (15.8)	1,598 (16.3)	1,562 (15.9)	0.005	0.004

### 3.2 Geographical distribution of DII

**Figure 2:**
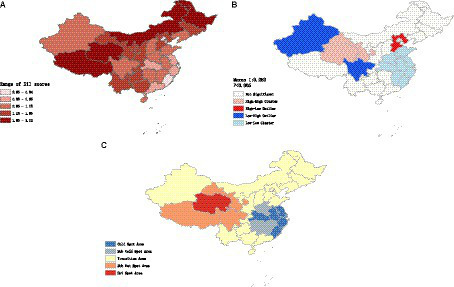
**(A)** Spatial distribution of the DII scores among adults aged 45 and above of China. **(B)** Clustering trend of DII scores among the Chinese middle aged and above population. **(C)** Hotspot and coldspot distribution of DII in Chinese middle aged and elders population.

Figure 2A showed the spatial distribution of the DII scores among adults aged 45 and above in China in 2015. Regions with higher DII scores are predominantly concentrated in northwest region, while regions with lower DII scores are mainly found in southeast coastal region. This indicates that in 2015, adults in northwest region had higher dietary inflammatory potential, whereas adults in southeast coastal region had lower dietary inflammatory potential.

According to the results of global spatial autocorrelation analysis (Figure 2B), a significant clustering trend of DII scores was observed (Moran I: 0.252, *p* < 0.05). This indicated that areas with high DII scores tended to be adjacent to other areas with high DII scores, while areas with low DII scores also tended to be adjacent to other areas with low DII scores.

Further analysis through local spatial autocorrelation revealed that high-high clustering areas of DII scores were primarily distributed in northwest region, where areas with high DII scores were surrounded by similarly high-scoring regions. Conversely, low-low clustering areas were primarily concentrated along the southeastern coastal regions, where areas with low DII scores were surrounded by similarly low-scoring regions.

The hotspot analysis of DII scores showed spatially clustered hotspot and coldspot areas (Figure 2C). Hotspot and sub-hotspot areas were primarily concentrated in the northwest region, whereas coldspot and sub-coldspot areas were mainly distributed along the southeastern coastal region.

Hotspot areas indicated regions with statistically significant high values of DII scores that were surrounded by neighboring areas with high values. This suggested a concentrated presence of higher DII in the northwest region, where the diet is mainly characterized by meat consumption and exhibits a high-salt feature. In contrast, coldspot areas indicated regions with statistically significant low values of DII scores that were surrounded by neighboring areas with low values. This suggested a clustering of lower DII in the southeastern coastal region, where the diet is relatively light and diverse in variety.

Overall, the southeastern coastal regions of China demonstrate lower dietary inflammatory potential, whereas the northwestern regions exhibit higher levels of dietary inflammation.

### 3.3 Associations of DII and hypertension

**Figure 3:**
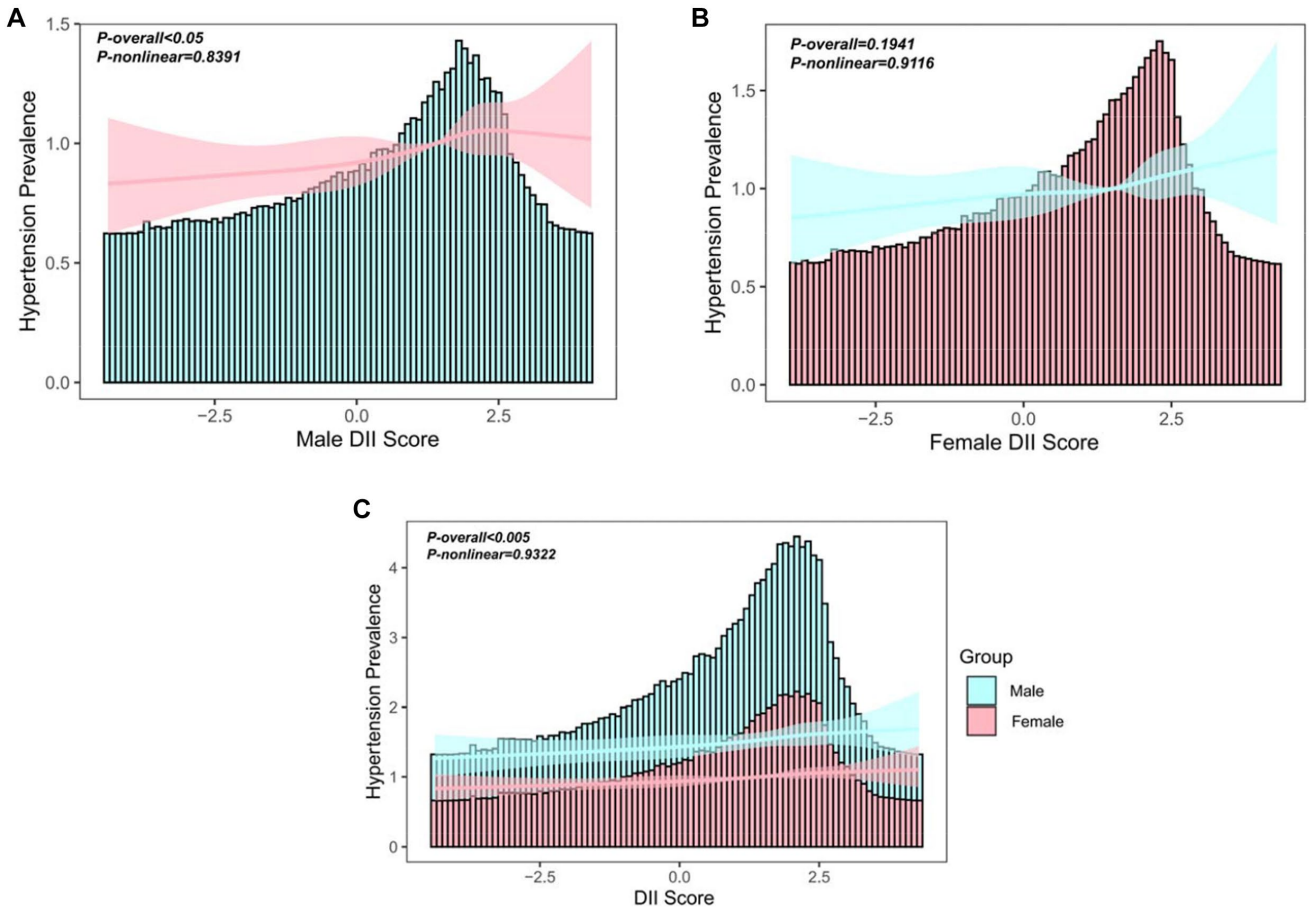
**(A)** The relationship between DII and hypertension in Chinese males aged 45 years and above. **(B)** The relationship between DII and hypertension in Chinese females aged 45 years and above. **(C)** The relationship between DII and hypertension in Chinese population aged 45 years and above.

Restricted cubic spline method was employed to further assess the dose–response relationship between DII scores and hypertension. Overall, a linear dose-response relationship was observed between DII scores and hypertension in the entire population and among males. Furthermore, this association may be more pronounced in males than in females (*p* < 0.05) (Figures 3A–C).

**Figure 4:**
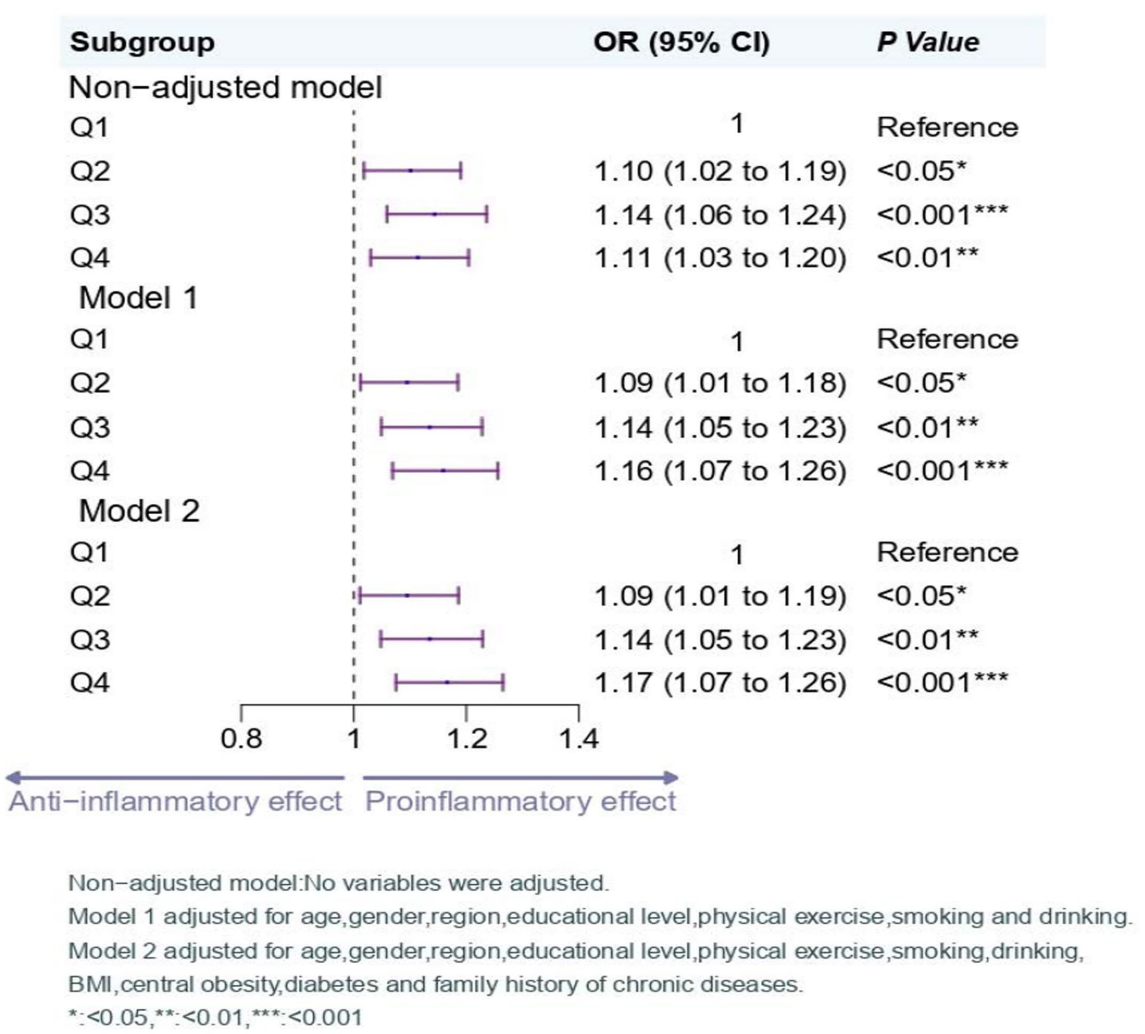
Logistic regression analysis on the association between the DII and hypertension.

According to the logistic regression modeling results of Figure 4, this study observed an association between DII scores and hypertension. In the unadjusted crude model, higher DII score groups showed a trend of increased risk for hypertension (Q2: OR 1.10, 95% CI 1.02–1.19; Q3: OR 1.14, 95% CI 1.06–1.24; Q4: OR 1.11, 95% CI 1.03–1.20) when compared with the lowest quartile of DII scores (Q1).

In model 1 and model 2, after adjusting for potential confounding factors, the similar association remained statistically significant. In both Model 1 and Model 2, the group with high DII (Q4) was found to have an increased risk of hypertension compared to the lowest quartile of DII scores (Q1), with an OR of 1.16 (95% CI 1.07–1.26) in Model 1 and an OR of 1.17 (95% CI 1.07–1.26) in Model 2.

### 3.4 Identification of key hypertension-related factors

**Figure 5:**
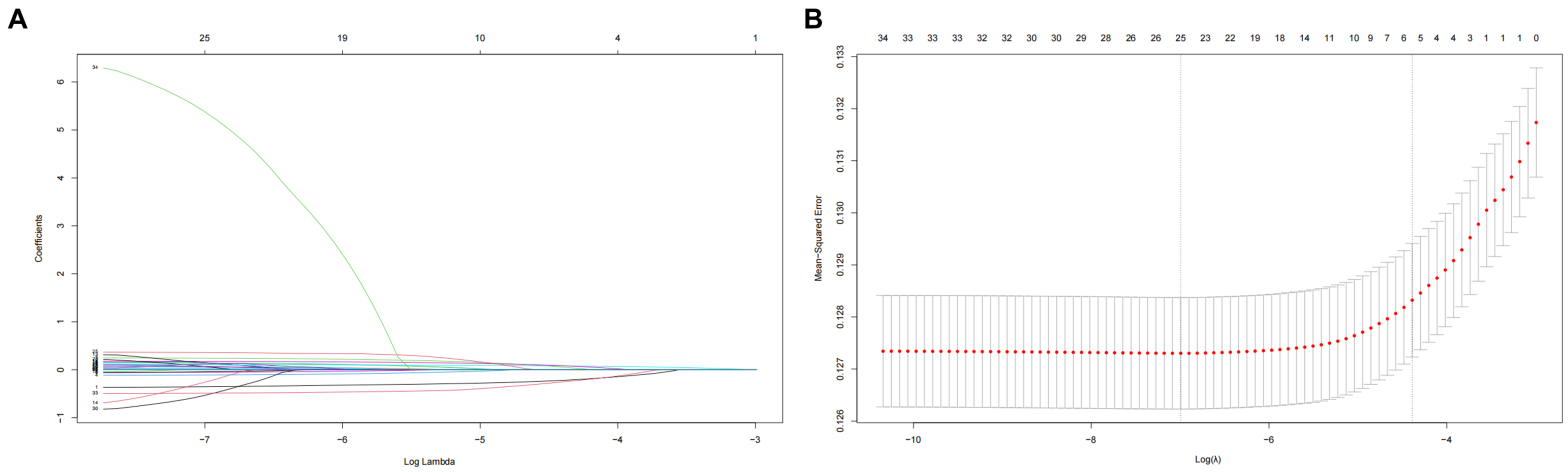
**(A)** Plot for coefficient shrinkage process of covariates and 23 components of DII. **(B)** Plot for 10-fold cross-validation of the LASSO regression model, least absolute shrinkage and selection operator.

**Figure 6:**
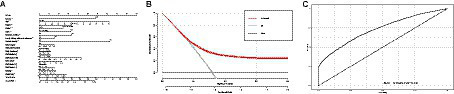
**(A)** Nomogram model based on the key factors identified by LASSO regression. **(B)** ROC curve for evaluating the predictive power for hypertension of the nomogram model. **(C)** DCA curve for evaluating the predictive power for hypertension of the nomogram model. ROC, receiver operating characteristic; DCA, decision curve analysis.

Utilizing LASSO regression analysis, we selected key indicators associated with hypertension along with covariates including age, gender, educational level, physical exercise, smoking, drinking, BMI, central obesity, diabetes, and family history of chronic diseases, as well as 23 components of the DII. Model evaluation and parameter selection were conducted through 10-fold cross-validation, leading to the identification of the lambda value (0.02233902) with the smallest deviation, thus determining the optimal model fit (Figures 5A,B). Subsequently, a set of 8 covariates (gender, age, region, nationality, alcohol consumption, diabetes, central obesity, and family history of chronic diseases) and 10 DII components (energy, carbohydrates, β-Carotene, Riboflavin, Vitamin B6, Vitamin C, Vitamin E, Folate, Mg, and MUFA) were pinpointed, laying the groundwork for the development of a nomogram model (Figure 6A). In addition, ROC and DCA curve analyses were employed to further evaluate the predictive capacity of the model and assess its impact on hypertension. The ROC curve analysis demonstrated an AUC of 0.732 (0.724–0.740) (Figure 6B), while the DCA results affirmed the model’s predictive robustness (Figure 6C). Notably, the full model exhibited a relatively high probability threshold, with the net benefit consistently exceeding 0 across nearly all probability thresholds, indicative of its strong predictive performance.

### 3.5 Sensitivity analysis

In this study, a sensitivity analysis was conducted on individuals under the age of 45, involving a total of 19,575 participants. Supplementary Table S1 provides a detailed overview of the demographic, physiological, and biochemical characteristics of these participants. The analysis revealed a weaker adverse impact of the DII on hypertension. Within the fully adjusted logistic regression model, only the Q3 and Q4 quartiles of DII displayed a significant association with hypertension (Q2: OR: 0.97, 95% CI: 0.84–1.13; Q3: OR: 1.10, 95% CI: 1.03–1.27; Q4: OR: 1.08, 95% CI: 1.01–1.25), consistent with the main analysis findings. This consistency supports the stability and reliability of the conclusions drawn in this study (Supplementary Table S2).

## 4 Discussion

### 4.1 Key findings of the study

This study is the first research on the association between DII and hypertension in the Chinese population aged 45 years and above using a national cross-sectional survey. A significant difference in DII scores between individuals with and without hypertension was observed, and a distinct spatial correlation in the geographical distribution of DII that higher DII scores were primarily observed in the northwest region of China, whereas lower DII scores were predominant in the southeast region. Those with higher DII scores (indicative of a pro-inflammatory diet) were positively correlated with an increased risk of hypertension. These findings remained consistent even after adjusting for confounding factors. Moreover, crucial factors related to hypertension were identified through Lasso regression analysis, leading to the establishment of nomogram model. The ROC and DCA curves exhibited strong predictive capabilities.

### 4.2 Association between DII and hypertension

The detrimental effects of elevated DII on health have been confirmed. A higher inflammatory potential in the diet may lead to the occurrence of inflammatory diseases and an increase in levels of inflammatory markers, including CRP, interleukin-6 (IL-6), interleukin-1 (IL-1), interleukin-2 (IL-2), tumor necrosis factor-alpha (TNF-α), interferon-gamma (IFN-γ), and vascular cell adhesion molecule (23–25). Elevated levels of CRP can heighten the risk of hypertension by reducing the expression and activation of nitric oxide synthase (26), increasing endothelin-1 synthesis (27), and impairing endothelial-dependent vasodilation (28). IL-6 can disrupt insulin signaling and activity, leading to the development of insulin resistance (29), which is one of the key mechanisms underlying hypertension. Additionally, inflammation is involved in the progression of atherosclerosis, which leads to vascular dysfunction. The inhibition of inflammation can attenuate vascular remodeling, ultimately reducing the risk of hypertension (30). Previous studies have also assessed the association between DII and the risk of hypertension or blood pressure changes. For instance, the SU.VI.MAX cohort study conducted in France found that higher DII scores were associated with an increased risk of metabolic syndrome as well as higher systolic and diastolic blood pressure (31). These findings aligned with the results of our study, where an increasing trend in systolic blood pressure (*p* for trend <0.0001) and diastolic blood pressure (*p* for trend = 0.003) were observed with an increase in DII scores. Moreover, a longitudinal study carried out in Australia specifically focusing on middle-aged and older women indicated that a pro-inflammatory diet may increase the risk of hypertension among women aged 52 and above (32). This study also found a rising proportion of women with increasing DII scores. This implies that females tend to have higher DII scores compared to males, indicating a potentially greater inflammatory capacity in their diet, which could elevate females’ susceptibility to inflammatory diseases. Previous studies have indicated that females tend to engage in less physical activity compared to males and are more prone to emotional overeating due to lifestyle stress and emotional factors (33). These factors may influence their dietary DII scores. However, it is worth noting that a study from Japan highlighted that women generally consume more anti-inflammatory foods (34, 35), indicating that the distribution of DII scores across genders may be influenced by national and regional variations.

However, restricted cubic splines indicated a more significant association between DII and hypertension in males compared to females suggesting that the relationship between DII and hypertension is stronger in the male population. Previous studies have shown that a significant association between DII and the risk of hypertension is only observed in middle-aged men, while no significant correlation is found in women (36). This difference may be attributed to higher DII scores leading to lower levels of male hormones. Studies in the United States have demonstrated that higher DII scores can increase the risk of testosterone deficiency in adult males and male adolescents (37, 38). Low testosterone concentration is considered an independent risk factor for male hypertension, as several population-based studies have indicated that increased testosterone levels can reduce the risk of hypertension in males (39–41). Testosterone may influence the occurrence of hypertension through its vasodilation effects on vascular and nonvascular smooth muscles (42, 43). Additionally, low testosterone levels in males are associated with insulin resistance and oxidative stress (44, 45), contributing to the development of hypertension.

### 4.3 Impact of regional variations in dietary patterns on DII scores

Previous studies have also indicated that a pro-inflammatory diet (characterized by higher DII scores), such as the Western diet, with its high fat, salt, and animal protein content, increases the risk of inflammatory diseases (46, 47). Conversely, diets with lower DII scores, such as the DASH and Mediterranean diets, have been associated to improved health outcomes (48). It has been reported that the DASH diet is associated with reduced levels of C-reactive protein (49). Variations in the dietary inflammatory potential across different dietary patterns may be attributed to differences in food composition and nutrient intake. In this study, employing lasso regression analysis, the DII components identified as significant contributors to hypertension included energy, carbohydrates, β-Carotene, Riboflavin, Vitamin B6, Vitamin C, Vitamin E, Folate, MUFA. Previous research has confirmed the roles of these nutrients in hypertension. The National Health and Nutrition Examination Survey (NHANES) project revealed that β-carotene (50), folate (51), and MUFA (52) may decrease the risk of hypertension. Moreover, meta-analysis findings suggest that Vitamin E can reduce systolic blood pressure (53). A 5% decrease in the risk of hypertension is linked to each additional intake of 100 mg/day of magnesium (54), whereas Vitamin B2, Vitamin C, Vitamin D, and folic acid do not exhibit antihypertensive effects (53). Hence, the differences in dietary inflammatory potential resulting from these distinct dietary patterns might lead to differences in inflammatory marker levels and the incidence of inflammatory diseases within the body, ultimately impacting the development of hypertension.

The variations in the regional distribution of the DII scores observed in this study may be explained by differences in typical dietary patterns and food environments across various regions. The southern region typically adheres to a Mediterranean dietary pattern, whereas the northwest region tends to adopt a Western dietary pattern. This discrepancy could potentially influence the variations in DII scores. The southeastern region represents the “Jiangnan dietary pattern” in China, which is considered relatively healthy. This dietary pattern involves the consumption of rapeseed oil and a higher proportion of polyunsaturated fats (particularly Omega-6). Similar to the Mediterranean dietary pattern, individuals following this pattern consume substantial amounts of vegetables and moderate quantities of fruits. However, the Jiangnan dietary pattern differs from the Mediterranean diet in terms of a higher intake of soy products (55). In contrast, the northwest region of China exhibits a striking contrast to the southeast region. Historically, the northwest region has been inhabited by nomadic ethnic groups, leading to a distinct lifestyle separate from the traditional agricultural practices. The primary means of sustenance in this region is animal husbandry, resulting in a relatively monotonous dietary structure with limited intake of fruits, vegetables and aquatic products (56, 57). In the northwest region, the main sources of protein are high intakes of meat and dairy products. Surveys have shown that meat and oil consumption in the northwest surpasses the highest recommended levels, while intake of eggs, seafood, dairy products, nuts, and legumes is below the minimum recommendations (58). Specifically in impoverished areas of the northwest, there is generally lower dietary diversity and micronutrient intake. Moreover, minority ethnic groups are more prone to micronutrient deficiencies compared to the Han ethnic group (59). This imbalance results in excessive fat and sodium intake exceeding the daily recommended levels in China (60). Notably, excessive salt intake has been identified as the foremost dietary risk factor for health issues in the northwest region of China based on data from the Global Burden of Disease project (61). Surveys have indicated that the standardized mortality rate for non-communicable diseases in western China is the highest nationwide (719–867/100,000), particularly for cardiovascular diseases (387.1–460.8/100,000). The estimated prevalence of hypertension in the western region (31.4%) also exceeds the national average in China (27.5%) (62). In contrast, the mortality rate and disease burden in the Jiangnan region are comparatively lower (63, 64).

To provide the national distribution of DII and examine the association between DII and hypertension, this study stands out as the first research study to utilize nationwide data. It offers a theoretical basis for disease prevention and dietary management in hypertension. Certain limitations are also exit in the study. Firstly, this was a cross-sectional study that could not account for temporality. Additionally, to avoid the influence of dietary changes on the results, hypertensive patients who adopted dietary intervention measures were not included in the study samples. Due to insufficient information on some parameters caused by objective conditions, 23 indicators were used to calculate DII scores for revealing the inflammatory potential of the diets, it is needed to expand the coverage of China’s food composition list for obtaining more accurate strength of association between DII and hypertension. Furthermore, because the dietary data in this study were obtained using food frequency questionnaires and dietary recall interviews administered by investigators to the participants, inherent problems like recall bias and reporting bias may be present during the survey. The conversion of food frequency questionnaires into daily dietary data could result in discrepancies with the actual daily dietary intake of the participants, possibly impacting the accuracy and validity of the dietary data.

## 5 Conclusion

The findings of this study indicated that regional disparities in dietary inflammatory potential were notable, with lower levels observed in the southeastern coastal regions of China and higher levels in the northwestern regions. There was a positive association between dietary inflammatory index and risk of hypertension in Chinese adults middle-aged and older. It might contribute to the prevention and management of hypertension through improving dietary habits, particularly by reducing the intake of inflammatory foods. Further research is needed to explore dietary patterns linked to reduced dietary inflammatory potential, offering dietary guidelines for preventing and managing hypertension.

## Data availability statement

The data analyzed in this study is subject to the following licenses/restrictions: the data presented in this study are not allowed to disclose. Requests to access these datasets should be directed to www.chinanutri.cn.

## Ethics statement

The studies involving humans were approved by Ethics Committee of the Chinese Center for Disease Control and Prevention (201519-B). The studies were conducted in accordance with the local legislation and institutional requirements. Written informed consent for participation was not required from the participants or the participants’ legal guardians/next of kin in accordance with the national legislation and institutional requirements.

## Author contributions

WD: Conceptualization, Writing – original draft. QM: Writing – review & editing. JZ: Writing – review & editing. ZL: Writing – review & editing. WG: Writing – review & editing. LZ: Writing – review & editing. PS: Conceptualization, Supervision, Writing – review & editing. GD: Writing – review & editing.
