# Supplementary material for: Geographic disparities of dietary inflammatory index and its association with hypertension in middle-aged and elders in China: results from a nationwide cross-sectional study
Source: Front Nutr. 2024 Mar 7;11:1355091. doi: 10.3389/fnut.2024.1355091 (PMC10955052; doi:10.3389/fnut.2024.1355091)
Supplement: Supplementary file 1 [file Data_Sheet_1.docx]

Supplementary Table 1: Baseline characteristics of the population under 45 years old

|  | ALL (N=19575) | Male(N=8900) | Female(N=10675) | P value |
| --- | --- | --- | --- | --- |
| Age (Median,Q1,Q3,y) | 36.0[29.2;41.4] | 36.1[29.2;41.5] | 35.9[29.2;41.4] | 0.951 |
| Region |  |  |  | 0.006 |
| urban | 8023(40.99) | 3553(39.92) | 4470(41.87) |  |
| rural | 11552(59.01) | 5347(60.08) | 6205(58.13) |  |
| Han ethnicity(n,%) | 16484(84.21) | 7462(83.84) | 9022(84.52) | 0.206 |
| Adequate physical activity (n,%) | 13644(69.70) | 6001(67.43) | 7643(71.60) | <0.001 |
| Educational level (n,%) |  |  |  | <0.001 |
| Below junior high school | 5573(28.47) | 2086(23.44) | 3487(32.67) |  |
| Junior high school | 10839(55.37) | 5423(60.93) | 5416(50.74) |  |
| Senior high school or above | 3163(16.16) | 1391(15.63) | 1772(16.60) |  |
| Marital status (n,%) |  |  |  | <0.001 |
| Other status | 2324(11.87) | 1483(16.66) | 841(7.88) |  |
| Having a partner | 17251(88.13) | 7417(83.34) | 9834(92.12) |  |
| Smoking (n,%) | 4886(24.96) | 4735(53.20) | 151(1.41) | <0.001 |
| Drinking (n,%) | 7779(39.74) | 5575(62.64) | 2204(20.65) | <0.001 |
| Sleep time (Median,Q1,Q3,h/d) | 8.00[7.00;8.50] | 8.00[7.00;8.00] | 8.00[7.00;8.50] | <0.001 |
| Sleep time group (n,%) |  |  |  | <0.001 |
| <6h/d | 483(2.47) | 235(2.64) | 248(2.32) |  |
| 6-8h/d | 13940(71.21) | 6548(73.57) | 7392(69.25) |  |
| >8h/d | 5152(26.32) | 2117(23.79) | 3035(28.43) |  |
| BMI (Median,Q1,Q3,,kg/m^2^) | 23.39[21.11;26.09] | 23.93[21.50;26.60] | 22.97[20.85;25.51] | <0.001 |
| BMI group(n,%) |  |  |  | <0.001 |
| Underweight | 972(4.97) | 373(4.19) | 599(5.61) |  |
| Normal weight | 10087(51.53) | 4150(46.63) | 5937(55.62) |  |
| Overweight | 5966(30.48) | 3017(33.90) | 2949(27.63) |  |
| Obesity | 2550(13.03) | 1360(15.28) | 1190(11.15) |  |
| Family history of chronic diseases (n,%) | 9288(47.45) | 4227(47.49) | 5061(47.41) | 0.918 |
| Central obesity (n,%) | 4846(24.76) | 2586(29.06) | 2260(21.17) | <0.001 |
| Hypertension (n,%) | 1775(9.07) | 1100(12.36) | 675(6.32) | <0.001 |
| Diabetes mellitus (n,%) | 654(3.34) | 396(4.45) | 258(2.42) | <0.001 |
| Fasting blood-glucose (Median,Q1,Q3, mmol/L) | 4.99[4.62;5.36] | 5.06[4.65;5.47] | 4.94[4.60;5.29] | <0.001 |
| Systolic blood pressure (Median,Q1,Q3, mmHg) | 121.33[112.67;131.33] | 125.33[117.67;135.00] | 117.67[109.67;126.67] | <0.001 |
| Diastolic blood pressure (Median,Q1,Q3, mmHg) | 75.33[69.00;82.33] | 78.00[71.67;85.00] | 73.00[67.00;79.67] | <0.001 |

Supplementary Table 2: Sensitivity analysis of the population aged 18 to 44 in the surveillance

| Subgroup | OR | 95%CI | P Value |
| --- | --- | --- | --- |
| No-adjusted Model |  |  |  |
| Q1 | 1 | 1 |  |
| Q2 | 0.96 | (0.84,1.11) | 0.60 |
| Q3 | 1.04 | (0.91,1.19) | 0.60 |
| Q4 | 0.98 | (0.85,1.12) | 0.75 |
| Model 1 |  |  |  |
| Q1 | 1 | 1 |  |
| Q2 | 0.95 | (0.83,1.1) | 0.49 |
| Q3 | 1.01 | (0.88,1.16) | 0.91 |
| Q4 | 0.98 | (0.85,1.14) | 0.82 |
| Model 2 |  |  |  |
| Q1 | 1 | 1 |  |
| Q2 | 0.97 | (0.84,1.13) | 0.72 |
| Q3 | 1.10 | (1.03,1.27) | <0.05 |
| Q4 | 1.08 | (1.01,1.25) | <0.05 |

*Non-adjusted model: No variables were adjusted. Model 1 adjusted for age, gender, region, educational level, physical exercise, smoking and drinking. Model 2 adjusted for age, gender, region, educational level, physical exercise, smoking, drinking, BMI, central obesity, diabetes and family history of chronic diseases.
